# Knowledge of oral health during pregnancy and associated factors among pregnant mothers attending antenatal care at South Omo Zone public hospitals, Southern Ethiopia, 2021

**DOI:** 10.1371/journal.pone.0273795

**Published:** 2022-08-29

**Authors:** Biresaw Wassihun, Abayineh Ayinalem, Kassaw Beyene

**Affiliations:** 1 Department of Midwifery, College of Health Sciences, Injibara University, Injibara, Ethiopia; 2 Department of Midwifery, College of Medicine and Health Sciences, Arba Minch University, Arba Minch, Ethiopia; Texas A&M University College Station, UNITED STATES

## Abstract

**Background:**

The awareness of women towards oral health during pregnancy is an important aspect of her general health. It can compromise pregnancy outcomes, as well as it may affect the newborn’s overall health. Every pregnant woman plays a critical role in shaping the oral hygiene habits adopted by her if she is aware of pregnancy-related oral health and result in good perinatal outcomes. This study aimed to assess knowledge of oral health during pregnancy and associated factors among pregnant mothers who attend antenatal care at South Omo Zone public hospitals, Southern Ethiopia, 2021.

**Methods:**

An institution-based cross-sectional study was conducted among pregnant women attending routine antenatal care from October 01 to November 30. A systematic random sampling method was conducted to select study participants. Data had been collected through face-to-face interviews using semi-structured questionnaires. Data were entered using epi data version 3.1 and exported to Statistical Package for the Social Sciences version 25 for data analysis. Descriptive statistics had calculated for each variable, and binary logistic regression analysis with 95% confidence intervals was carried out to assess the factors associated with the outcome variables. Variables with P-value less than 0.05 were used to declare statistical significance.

**Result:**

Overall good knowledge of pregnant mothers regarding oral health was 34.1% with 95% CI, (32.76, 35.44). Having access to health facility (AOR = 2.60, 95% CI, 1.44, 4.70), having the educational status of secondary and above (AOR 1.37, 95% CI, 1.44, 4.31), having the educational status of primary education (AOR = 0.43, 95% CI, 0.20, 0.92), having a median income of > = 1500 Ethiopian birr (AOR = 0.41, 95% CI, 0.23, 0.72), being governmental employee (AOR = 0.11, 95% CI, 0.03, 0.41), received oral health hygiene counseling during pregnancy (AOR = 1.33, 95% CI, 1.62, 2.84) were significantly associated with good knowledge of oral health of pregnant mothers.

**Conclusion:**

This study showed that the knowledge of pregnant mothers about oral health was 34.1%. Educational status, monthly income, occupation, access to health services, and receiving counseling on oral hygiene at antenatal care were some factors associated with good knowledge of oral health during pregnancy. Therefore strengthening counseling during antenatal care, improving access to a health facility, improving educational status, monthly income, and being government employee are crucial to enhance knowledge of women towards oral health during pregnancy.

## Background

Oral health is a key indicator of overall health, well-being, and quality of life. The World Health Organization defines oral health as “a state of being free from chronic mouth and facial pain, oral and throat cancer, oral infection and sores, periodontal (gum) disease, tooth decay, tooth loss, and other diseases and disorders that limit an individual capacity in biting, chewing, smiling, speaking and psychosocial wellbeing [1]. In the oral cavity, various pathologies have been reported among pregnant women. The exaggerated inflammatory response of the gums to bacterial plaque known as pregnancy gingivitis has been attributed to the increased secretion of gestational hormones (especially estrogen and progesterone) during pregnancy. Proper nutrition and a healthy lifestyle also play a vital role in the general wellbeing of the mother [2, 3].

There are many common oral problems in pregnancy such as pregnancy gingivitis, benign gingival lesions, tooth mobility, tooth erosion, dental caries, and periodontitis [2]. The increased consumption of refined carbohydrates will provide a suitable substrate for carcinogenic bacteria and may predispose to increased tooth decay in some individuals. The frequent vomiting associated with pregnancy in some women is also known to predispose to the development of dental erosion [3].

Oral health is an important issue to the general health of both the woman and her infant [4]. Evidence showed that insufficient oral health care during pregnancy can have negative outcomes for both mothers and their newborns [5].

Oral health is one of the pregnancy-related issues in Ethiopia, the practice of oral hygiene lacks proper attention and care, whereby the habit of tooth brushing is found to be a minimum [6, 7].

The study finding in Shashemene, Ethiopia revealed only 34.6% of pregnant women had good knowledge towards their oral health during pregnancy [8].

Pregnancy-associated gingivitis is similar to common plaque-related gingivitis but with more severity; the severity being correlated with blood steroid hormone levels [4]. Periodontal disease during pregnancy has been criticized to be associated with adverse perinatal outcomes, including preeclampsia, preterm delivery, low birth weight, increased fetal death, and newborn care time in the neonatal care unit [9–11]. Therefore, women should be given proper oral hygiene and oral health preventive services before, during, and even after childbirth [12].

Oral health in pregnancy is important to the health of the pregnant woman, and good oral health plays a role in the outcome of pregnancy. Therefore this study was intended to assess knowledge of oral health of pregnant women attending ANC and associated factors at South Omo Zone public hospitals, Southern Ethiopia.

## Methods and materials

### Study area and design

A facility based cross sectional study design was conducted in South Omo Zone public hospitals from October 01 to November 30,2021. South Omo Zone is located 820 kilometers from Addis Ababa the capital of Ethiopia, and 530 km away from Hawassa the capital city of SNNP. According to Population projection values of 2017, the zone had a total population of 722,955 of whom 360,517 are males and 362,438 are females. 93.07% of the population is rural Dowler. South Omo is bordered on the south by Kenya, on the southwest by South Sudan, on the west by Bench Maji, on the northwest by Keffa, on the north by Konta, Gamo Gofa, and Basketo, on the northeast by Dirashe and Konso, and on the east by the Oromia Region. South Omo Zone is structured of ten districts and one administrative town. The administrative center of South Omo is Jinka. South Omo Zone has only two hospitals which are Jinka general hospital and Gather primary hospital which served around 1.2 million people in the catchment area.

### Populations

All pregnant women who attend antenatal care services at South Omo Zone public hospitals were the sources population. Pregnant women who attend antenatal care services at South Omo Zone public hospitals during the study periods were the study populations. Those women who are critically ill and already oral health treatment were excluded from the study.

### Sample size determination

The minimum required sample size was determined by using single population proportion formula from a previous study done in Shashemenie, Ethiopia by considering the following formula;

$$n=\frac{Z^{2}P(1-P)}{d^{2}}$$

$$n=\frac{{\left(1.96\right)}^{2}*0.346(1-0.346)}{{\left(0.05\right)}^{2}}\;n=348$$

Where

n = calculated minimum sample size

Z_∝/2_ = standard normal distribution curve critical value for 95% CI = 1.96

d = the acceptable margin of error (precision) = 0.05

P = Assumed proportion of knowledge of pregnant women at ANC is 34.6% [8], which was taken from a study done in Shashemenie, Ethiopia.

The largest sample size was 348, and 10% of the total sample size was added to compensate non-response rate and the final sample size was 384.

### Sampling technique and procedures

The distribution of the sample to each hospital was made proportionally based on the number of women who get antenatal care at each facility in the two months preceding the data collection period.

Study participants at each hospital were carefully chosen by systematic random sampling during the data collection period until the required sample size was attained.

The sampling interval k = 2 was calculated by dividing the source population of pregnant women by the total sample size and this interval was used in both hospitals to select study subjects.

Therefore, the first women from each hospital were selected by lottery method. Then every other woman was interviewed.

### Study variables

#### Dependent variables

Knowledge of oral health during pregnancy.

#### Independent variables

*Socio-demographic variables*. Age, marital status, place of residence, family income, educational status of the mothers, occupation, Religion.

*Obstetric factors*. Gravidity, parity, gestational age, Antenatal care, periodontitis, gingivitis, and dental caries.

*Health facility-related factors*. Attitude of health worker, waiting time, lack of space in ANC, lack of skilled health care provider.

### Operational and term definition

#### Oral health

A state of being free from any pain originating from mouth and face, oral and throat cancer, oral infection and sores, periodontal disease, tooth decay, tooth loss, and other diseases and disorders that limit an individual’s capacity in biting, chewing, smiling, speaking, and psychosocial wellbeing.

#### Oral health knowledge

The capacity to obtain, communicate, process, and understand basic oral health information and services to make appropriate oral health decisions.

#### Good knowledge

If the respondents answer greater than or equal to the mean value of knowledge questions.

#### Poor knowledge

If the respondents answer less than the mean value of knowledge questions.

### Data collection tools

Adapted and semi-structured questionnaires were used to collect data from the antenatal women in the ante natal clinic of each health facilities. Trained interviewers were administered the questionnaire after obtaining written consent from each antenatal woman. The questionnaire contained information about socio-demographic factors, knowledge of pregnant mothers towards oral health, health facility-related factors, and obstetric-related factors.

The questionnaire was developed in the English language and then translated into Amharic. The Amharic version of the questionnaire was translated back into the English language to confirm the correct and precise interpretation of the questionnaire. Four BSc midwives data collectors and two MSc midwives supervisors were recruited for data collection.

### Data quality control

The pretest was done on 5% of the sample size (by 20 questionnaires) to ensure its consistency and validity then correction was made accordingly before the actual data collection. The one-day training was given to data collectors and supervisors about the methodology and questionnaires by the principal investigator.

During the data collection period, study participants were informed about the purpose and the importance of the study. The collected data were checked for completeness and consistencies by trained supervisors and investigators on daily basis and immediate action was taken accordingly. After data collection, the collected data were rechecked for completeness and consistency by the investigator.

### Data processing and analysis

The collected data was patterned by the principal investigator, and then data were coded, entered, and cleaned using Epi Data version 4.4.3.1 software and finally exported into SPSS version 25.0 software for analysis. Descriptive statistics of different variables were determined and the result was presented in tables, charts, and graphs using summary measures such as percentages mean, and median. Binary logistic regression was used for the analysis of the outcome variable.

A Hosmer-Lemeshow test was done to test for model fitness. Bivariate analysis was carried out to identify the factors associated with the knowledge of oral health during pregnancy.

All variables were taken into the multivariable model by considering a p-value of < 0.25 to see the independent effect of each variable on the outcome variable. The multi-co-linearity test was carried out to see the correlation between independent variables.

Finally, the result of bivariate and multivariable logistic regression analysis was presented in a crude and adjusted odds ratio with 95% confidence intervals. All tests were two-sided and P< 0.05 was considered statistically significant.

### Ethical clearance

Ethical clearance was obtained from the Institutional Research Ethics review board of the college of medicine and health science, Arba Minch University (IRB/21). Approval was obtained from the managers of each health institution. All measures were employed to maintain human rights including written consent, written assent form was prepared for minors (age less than 18 years) so that those minors and their parents/guardians were put their signature, the right to participate in the study, the right to privacy and confidentiality, and the right to prevention from any form of harm. All participants were informed about the objectives of the study and that their participation was voluntary. It was also clearly stated to the participants that the information they provided was for research purposes and strictly confidential. Data were collected after the women got antenatal care.

## Results

### Socio-demographic characteristics

A total of 384 pregnant women were interviewed with a 100% response rate. The mean age of pregnant mothers was 27.61, (SD ±5.16) with an age range of 16 and 42 years. Majority of the respondents 150 (39.1%) were found in the age group of 25–29 years followed by 88(22.9%) and which were found in the age group 30–34 years. Most of the women 367(95.6%) were married and 13(3.4%) were divorced. Regarding ethnicity 163(42.4%) of them was Ari, which is followed by Amhara (33, 3%) Ethnicity. Regarding education 162(42.2%) of the respondents have attended primary school. Concerning occupation 215(56%) were housewives and 87(22.7%) were farmers. Regarding income, only 50(13%) of the study participants had an income of (1001–2500 ETB). The majority of the respondents 236(61.5%) were found in the urban area (**Table 1**).

**Table 1:** Socio-demographic characteristics of pregnant mothers attending antenatal care at South Omo Zone hospitals, Southern Ethiopia, 2021.

Variables		frequency(n =)	Percentage (%)
Age	15–19	27	7
	20–24	76	19.8
	25–29	150	39.1
	30–34	88	22.9
	35–39	38	9.9
	40–44	5	1.3
Marital status	Single	3	0.8
	Married	367	95.6
	Divorced	13	3.4
	Widowed	1	0.3
Religion	Muslim	62	16.1
	Orthodox	157	40.9
	Protestant	163	42.4
	Others	2	0.5
Ethnicity	Ari	163	42.4
	Bena	62	16.1
	Mursi	29	7.6
	Amhara	128	33.3
	Others	2	0.5
Educational status	No formal education	77	20.1
	Read and write	83	21.6
	Primary	612	42.2
	Secondary and above	62	16.1
Occupation	Farmer	87	22.7
	Merchant	35	9.1
	Housewife	215	56
	Government employee	47	12.2
Average monthly income	<1000	75	19.5
	1001–2500	50	13
	2501–5000	40	10.4
	> = 5000	2	0.5
	No income	217	56.5
Residency	Urban	236	61.5
	Rural	148	38.5

### Obstetric characteristics of the study participants

**Fig 1:**
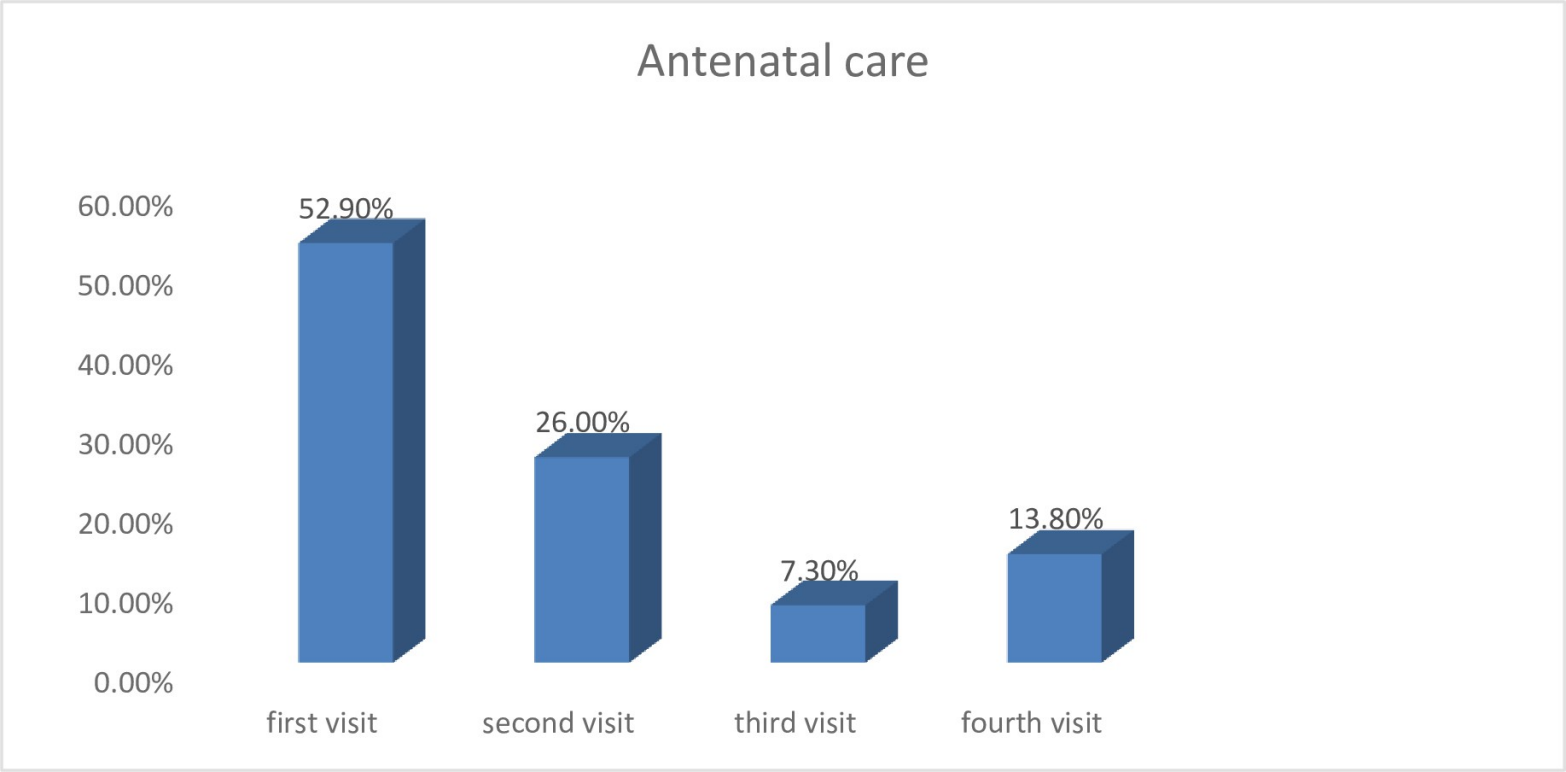
Antenatal care status of pregnant mothers at South Omo Zone hospitals, Southern Ethiopia, 2021.

Among the pregnant mothers, 98(25.5%) were primipara and 286(74.5%) were multipara. From multiparous women, 73(74.5%) of mothers have two live births. Among multiparous mothers, 98(25.5%) had a history of oral health problem during a previous pregnancy. From multiparous 32(11.2%) have previous home delivery and 254(88.8%) of them were delivered in the health institutions. Among pregnant mothers who come to ANC 95(24.7%) were in the first trimester, 145(37.8%) were in the second trimester and 144(37.5%) were in the third trimester of pregnancy. Among pregnant mothers 203(52.9%) had first visited, 100(26%) had the second visit, 28(7.3%) had a third visit and only 53(13.8%) of them had a fourth visit. Among pregnant mothers who came to ANC 17(4.4%) have the medical disease and among those 2(11.8%) had anemia, 4(23.5%) had diabetes mellitus, 8(47.1%) had hypertension and 3(13.6%) had HIV AIDS (**Fig 1**).

### Health facility-related factors

**Fig 2:**
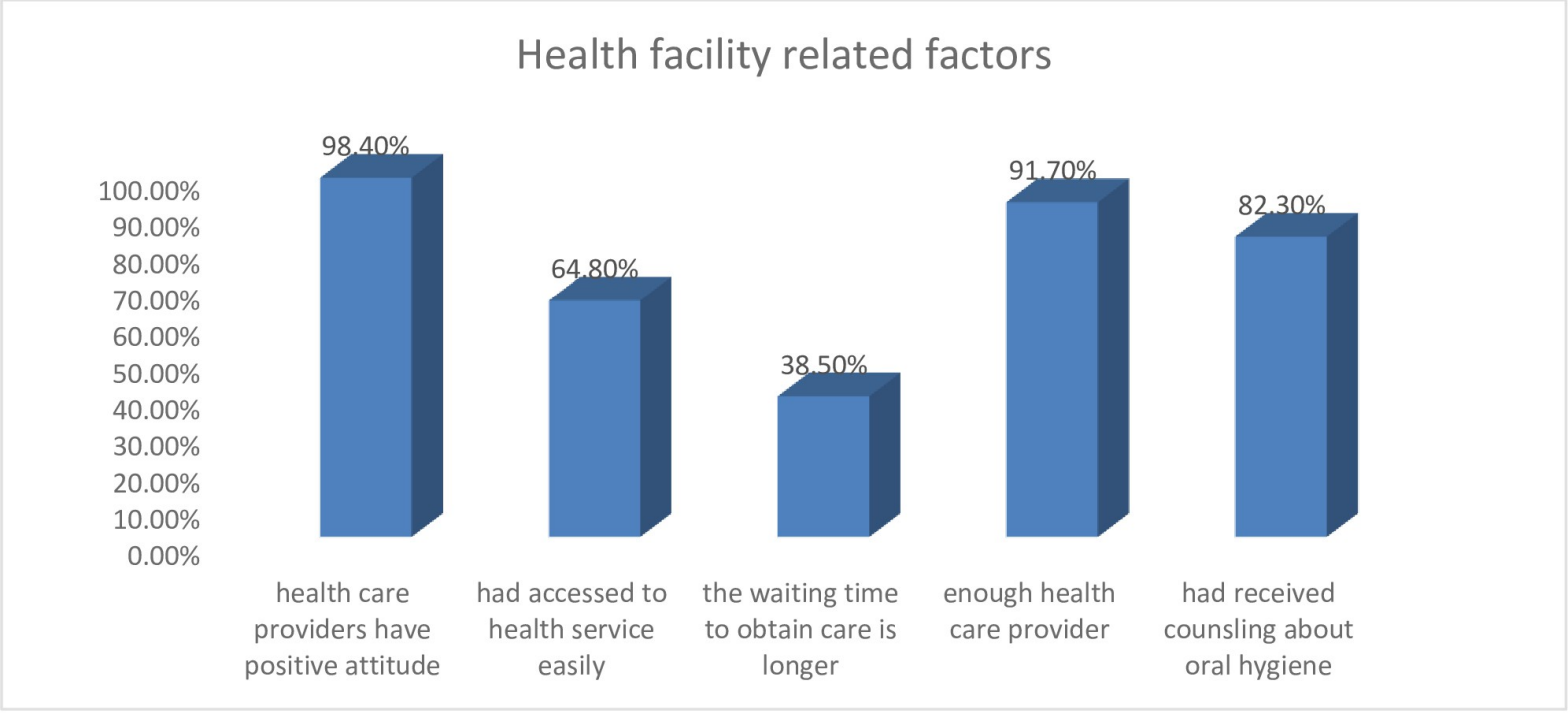
Health facility related factors associated with knowledge of pregnant mothers attending antenatal care in South Omo Zone hospitals, Southern Ethiopia, 2021.

Among the pregnant mothers, 378(98.4%) thought that health care providers have a positive attitude while providing care, 249(64.8%) had access to health services easily, 148(38.5%) thought that the waiting time to obtain care is longer, 352(91.7%) thought that there were enough skilled health care provider and 316(82.3%) had received counseling on oral hygiene (**Fig 2**).

### Knowledge of pregnant women about oral health

**Fig 3:**
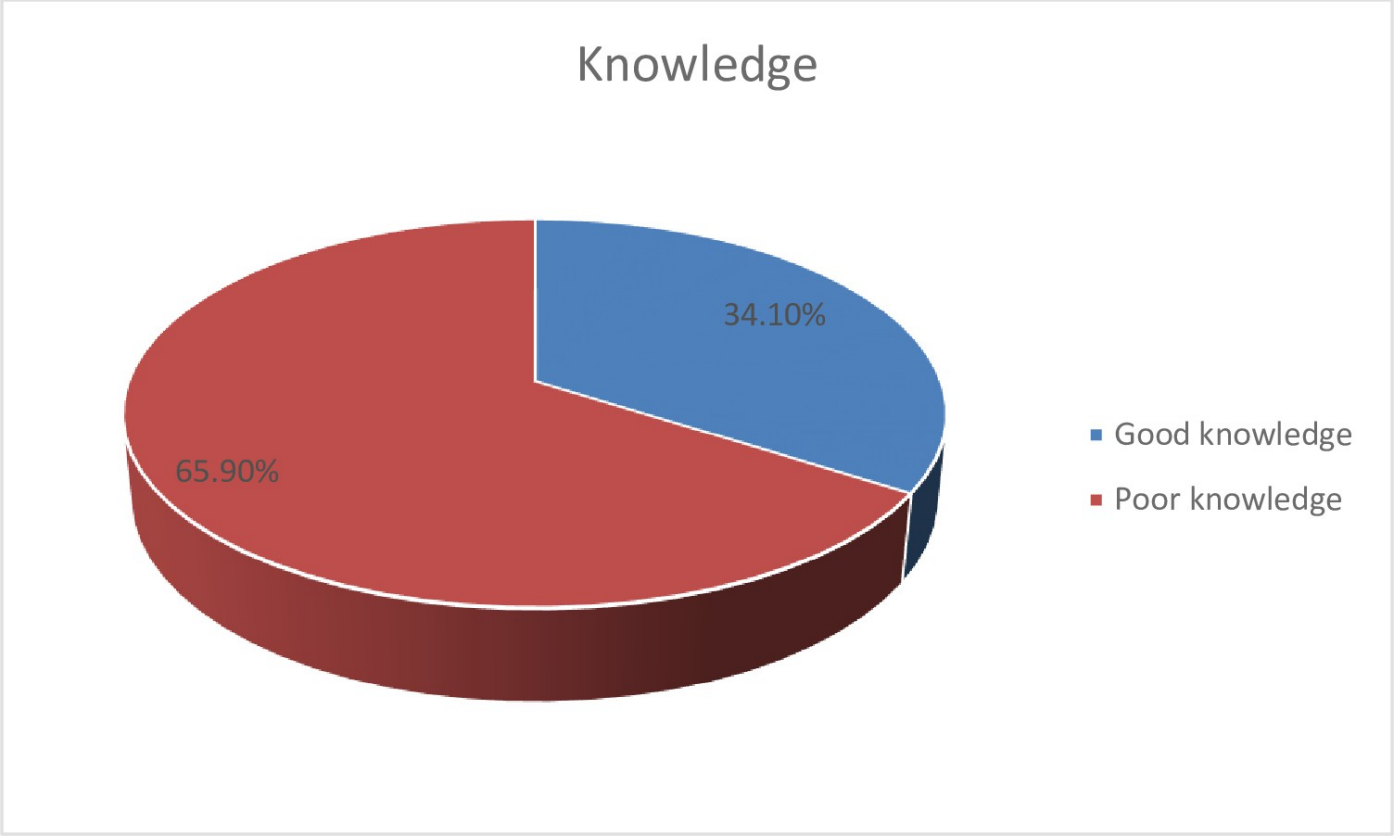
Knowledge of pregnant mothers about oral health during pregnancy attending antenatal care at South Omo Zone hospitals, Southern Ethiopia, 2021.

Overall, more than one third (34.1%) of pregnant women had good knowledge regarding oral health during pregnancy while 251(65.9%) of them had poor knowledge about oral health (**Fig 3**).

### Bivariate and multivariable logistic regression

To determine the association between knowledge of oral health during pregnancy with different factors, the following explanatory variables were checked against outcome variables

On bivariate analysis, educational level, family income, residency, occupation, gestational age/ trimester, access to health facility and received counseling on oral health have a significant association with knowledge of oral health during pregnancy.

To control the effects of confounder multivariable analysis was carried out. On multivariable analysis, access to a health facility, educational status of secondary and above, having better family income, being governmental employee and receive oral health hygiene counseling have a statistically significant association with knowledge of oral health.

Those mothers who have access to a health facility during pregnancy were 2.6 times more likely to be knowledgeable as compared to those mothers who have no access to a health facility during pregnancy (AOR = 2.6 95% CI, 1.44, 4.70). Those pregnant mothers having an educational status of secondary and above (AOR 1.37, 95% CI, 1.44, 4.31) were 1.37 times more likely to be knowledgeable on oral health during pregnancy than those mothers having an educational status of no formal education. Those mothers having the educational status of primary education (AOR = 0.43, 95% CI, 0.20, 0.92) were 43% times less knowledgeable than counterparts. Pregnant women who had a median family income of > = 1500 Ethiopia birr (AOR = 0.41, 95% CI, 0.23, 0.72) were 41% more likely knowledgeable than women who had less median family income. Pregnant mothers who are governmental employee (AOR = 0.11, 95% CI, 0.03, 0.41), were 11% more likely to be knowledgeable than women are merchant and farmers. Women who received oral health hygiene counseling during pregnancy (AOR = 1.33, 95% CI, 1.62, 2.84) were 1.33 times more likely to be knowledgeable than pregnant women who did not received health care hygiene counseling (Table 2).

**Table 2:** Bivariate and multivariable logistic regression on knowledge of pregnant mothers attending antenatal care at South Omo Zone Hospitals, Southern Ethiopia, 2021.

Variable		Knowledge of oral health		COR 95%CI	AOR 95%CI
		Good	poor		
Educational level	No formal education	50	27	1	1
	Primary	61	184	0.18(0.10–0.31)	0.43(0.20–0.92)
	Secondary and above	20	42	0.26(0.13–0.52)	1.37(1.44–4.31)*
Family monthly income	≤1500	40	51	1	1
	>1500	91	202	0.57(0.35–0.93)	0.41(0.23–0.72)*
Occupation	Farmer	60	27	1	1
	Merchant	61	189	015(0.09–0.23)	0.31(0.14–0.71)*
	Government employee	10	37	0.12(0.05–0.28)	0.11(0.03–0.41)*
Residency	Rural	76	72	1	1
	Urban	55	181	0.29(0.19–0.45)	0.53(0.27–1.06)
Trimester	First trimester	26	69	1	1
	Second trimester	45	100	1.19(0.67–2.12)	0.89(0.47–1.73)
	Third trimester	60	84	1.89(1.08–3.32)	0.99(0.50–1.97)
Access to a health facility	Yes	105	144	3.06(1.86–5.02)	2.60(1.44–4.70)*
	No	26	109	1	1
Received counseling on oral hygiene	Yes	116	200	2.05(1.11–3.80)	1.33(1.62–2.84)*
	No	15	53	1	1

* = p-value <0.05, CI = Confidence Interval, COR = Crude Odds Ratio, AOR = Adjusted Odds Ratio

## Discussions

The overall percentage of good knowledge of the pregnant woman regarding oral health during pregnancy in our study is 34.1% e. This is comparable to the finding of good knowledge that (34.6%) was reported from the Shashemene, Ethiopia [12]. The finding of this study is smaller than the finding reported from Qassim province, Kingdom of Saudi Arabia, and India which was 44.32% and 52.4% respectively [13, 14]. The discrepancy might be due to sociocultural, socio-demographic characteristics of study participants and study period. In the current study, pregnant mothers receiving counseling during antenatal care about oral health were 1.33 times higher than those who don’t receive counseling during antenatal care. This is consistent with a study conducted in Spain with the finding that pregnant women who assisted to talk about oral health promotion had better knowledge [15]. In this finding, pregnant mothers with the educational status of secondary and above were 1.37 times more likely knowledgeable on oral health during pregnancy than those who have no formal education, which is consistent with the study conducted in Spain with the finding that the pregnant women with higher education had a better knowledge of oral health compared with those who had primary and no educations [15]. This may be due to women that attend higher education may have better awareness about their general health status including oral health. In this study finding parity has no association with knowledge of oral health, but according to the study done in Brazil, pregnant women who already had children had better knowledge than those who were in their first pregnancy [16]. This might be attributed due to the fact that in our study even if women are multiparous, they are not knowledgeable because they are not informed about oral health during their pregnancy at antenatal clinic.

In these findings, pregnant mothers with educational status of primary were 43% times more likely knowledgeable than counterparts. A study conducted in East Wollega Ethiopia showed that relative to women with no education, women with primary education had significantly greater odds of good knowledge during pregnancy [17].

## Conclusions

This study showed that the knowledge of pregnant mothers about oral health was 34.1%. Educational status, monthly income, occupation, access to health services, and receiving counseling on oral hygiene at ANC were factors associated with knowledge of oral health during pregnancy. Therefore strengthening counseling during ANC, improving access to the health facility, improving educational status, monthly income, and occupation are some crucial events to increase knowledge of oral health during pregnancy. The hospital administrators and health care providers should make efforts to increase community-based health education, awareness creation and improve better access to information for mothers regarding oral health care.

## Supporting information

S1 File Data collection tool. (DOCX)

S2 File The dataset used for this study. (SAV)

## List of abbreviations

ANC: Ante Natal Care
AOR: Adjusted Odd Ratio
CI: Confidence Interval
WHO: World Health Organization
SNNPRS: Southern Nation Nationalities and Peoples Regional state
