# Supplementary material for: Knowledge of oral health during pregnancy and associated factors among pregnant mothers attending antenatal care at South Omo Zone public hospitals, Southern Ethiopia, 2021
Source: PLoS One. 2022 Aug 29;17(8):e0273795. doi: 10.1371/journal.pone.0273795 (PMC9423605; doi:10.1371/journal.pone.0273795)
Supplement: S1 File — (DOCX) [file pone.0273795.s001.docx]

**ENGLISH VERSION QUESTIONAIRE**

| Part 1: socio demographic characteristics of pregnant women attending ANC | | | |
| --- | --- | --- | --- |
| Q.no | Questions | Choice for response | Code |
| 1.1 | Age |  |  |
| 1.2 | Marital status | 1. Single 2. Married  3. Divorced 4. Widowed |  |
| 1.3 | Religion | 1. Muslim 2. Orthodox  3. Protestant 4. Others----- |  |
| 1.4 | Ethnicity | 1. Ari 2. Bena 3. Mursi 4. Amhara 5. Other specify -------- |  |
| 1.5 | Educational status | 1. No formal education  2. Read and write  3. Primary education  4. Secondary and above |  |
| 1.6 | Occupation | 1. Farmer 2. Merchant  3. Housewife 4. Government employed 5. If Other specify----- |  |
| 1.7 | Income per month | ------------ETB |  |
| 1.8 | Residence | 1. Rural 2. Urban |  |

| **Part 2: Knowledge of pregnant women about oral health** | | | |  |
| --- | --- | --- | --- | --- |
| 2.1 | Do heard what oral health means during pregnancy? | 1. Yes 2. No   If no go to Q2.3 | |  |
| 2.2 | If yes, the source of information? | 1. Mas-media 2. Health care provider 3. Others specify--------- | |  |
| 2.3 | Do you know that tooth cleaning is more necessary during pregnancy | 1. Yes 2. No 3. Don,t know | |  |
| 2.4 | Do you know that tooth should be cleaned at least twice a day | 1. Yes 2. No 3. Don’t know | |  |
| 2.3 | Do you know that tooth should be cleaned using tooth brush with tooth paste for at least 2-3 minutes | 1. Yes 2. No 3. Don’t know | |  |
| 2.4 | Do you know that not keeping oral hygiene can lead to gum and tooth disease | 1. Yes 2. No 3. Don’t know | |  |
| 2.5 | Do you know that gum disease can cause bad oral breath | 1. Yes 2. No 3. Don,t know | |  |
| 2.6 | Do you know that periodontitis could result in tooth loss | 1. Yes 2. No 3. Don’t know | |  |
| 2.7 | Do you know that gingivitis during pregnancy could lead to LBW and PTB | 1. Yes 2. No 3. Don’t know | |  |
| 2.8 | Do you know that pregnant women may be susceptible to gingivitis | 1. Yes 2. No 3. Don,t know | |  |
| 2.9 | Gingivitis can cause red, swollen gums that will bleed easily | 1. Yes 2. No 3. Don’t know | |  |
| 2.10 | Gingivitis can be treated with careful brushing and flossing at home | 1. Yes 2. No 3. Don’t know | |  |
| **Part Three: obstetric factors characteristics** | | | | |
| 3.1 | Parity | | 1. Primipara  2. Mulltipara  If primipara, go to Q3.5 |  |
| 3.2 | If multipara, how many live births do you have? | | 1. Two 2. More than two |  |
| 3.3 | If multipara, did you have faced oral health problem in the previous pregnancy? | | 1. Yes 2. No |  |
| 3.4 | If multipara, place of previous delivery? | | 1. Home 2. Institutional |  |
| 3.5 | Gestational age? | | 1. 1^st^ trimester 2. 2^nd^ trimester 3. 3^rd^ trimester |  |
| 3.6 | Antenatal care follow up | | 1. One times 2. Two times 3. Three times 4. Four times |  |
| 3.7 | Do you have any medical diseases? | | 1. Yes 2. No |  |
| 3.8 | If yes for question 4.3 list the diseases | |  |  |
| **Part four: health facility related characteristics** | | | | |
| 4.1 | Do think that health care providers have positive attitude while providing care? | 1. Yes 2. No | |  |
| 4.2 | Do you access the health care service easily /without difficulty? | 1. Yes 2. No | |  |
| 4.3 | Do you think that the waiting time to obtain care is longer? | 1. Yes 2. No | |  |
| 4.4 | Do you think that there are enough skilled health care providers? | 1. Yes 2. No | |  |
| 4.5 | Do you receive counsling about oral hygine during Atenatal care? | 1. Yes 2. No | |  |
